# Study on a Fault Mitigation Scheme for Rub-Impact of an Aero-Engine Based on NiTi Wires

**DOI:** 10.3390/s22051796

**Published:** 2022-02-24

**Authors:** Qiang Pan, Tian He, Wendong Liu, Xiaofeng Liu, Haibing Chen

**Affiliations:** 1 School of Transportation Science and Engineering, Beihang University, Beijing 100191, China; panqiang@buaa.edu.cn (Q.P.); liuxf@buaa.edu.cn (X.L.); aw_chenhb@buaa.edu.cn (H.C.); 2 AECC Shenyang Engine Research Institute, Shenyang 110000, China; lwdbuaa@163.com

**Keywords:** rub-impact, tip clearance, shape memory alloy, aero-engine, fault mitigation

## Abstract

The aim of this study was to solve the frequently occurring rotor-stator rub-impact fault in aero-engines without causing a significant reduction in efficiency. We proposed a fault mitigation scheme, using shape memory alloy (SMA) wire, whereby the tip clearance between the rotor and the stator is adjusted. In this scheme, an acoustic emission (AE) sensor is utilized to monitor the rub-impact fault. An active control actuator is designed with pre-strained two-way SMA wires, driven by an electric current via an Arduino control board, to mitigate the rub-impact fault once it occurs. In order to investigate the feasibility of the proposed scheme, a series of tests on the material properties of NiTi wires, including heating response rate, ultimate strain, free recovery rate, and restoring force, were carried out. A prototype of the actuator was designed, manufactured, and tested under various conditions. The experimental result verifies that the proposed scheme has the potential to mitigate or eliminate the rotor-stator rub-impact fault in aero-engines.

## 1. Introduction

The efficiency of a rotating machine such as an aero-engine is strongly dependent on the tip clearance between the stationary and rotating parts [1]. In order to improve the efficiency of an aero-engine, the clearance should be designed as small as possible. It has been reported that a 0.0254 mm reduction in the tip clearance of a high-pressure turbine may lead to a decrease of 0.1 percent in specific fuel consumption [2,3]. However, minimizing the clearance is usually associated with undesired rub-impact phenomena occurring between the rotor and the casing due to mechanical, aerodynamic excitation, or thermal gradience during engine operation [4,5]. This leads to material or structural damage, e.g., plastic deformations, changes in the microstructure on the blade tips, crack initiation, and the break out of liner material at the rubbing zone [6], and, sometimes, catastrophic accidents [7].

Researchers have strived to understand the rub-impact fault, for example, exploring the fault mechanism [8,9,10], the fault feature extraction method [11,12,13], source localization [14,15], and intelligent fault diagnosis [16,17], etc. These studies contributed to the development of rub-impact fault diagnosis and to practical applications. However, most studies focused on how to monitor the occurrence of a fault and analyze its type or location; few were concerned with the mitigation or elimination of the rub-impact phenomenon. Unfortunately, rub-impact is a fault which may induce disastrous accidents, if it cannot be mitigated in time.

In order to protect the rotor from rub-impact from the casing of an aero-engine, a feasible solution is to adjust the tip clearance when it occurs. The most popular scheme to achieve this that is used in the design of aero-engines is based on the active clearance control (ACC) method [18]. For example, Bucaro et al. designed a new gas turbine engine thermal control device to improve the control efficiency in terms of the active thermal control system [19]. Tillman et al. proposed a system and method of active thermal control that includes processing aircraft data when the aircraft is flying at altitude cruise conditions [20]. Decastro et al. used two actuators consisting of electrohydraulic servo valves and piezoelectric stacks to adjust the shroud in a high-pressure turbine section [21]. However, ACC is generally used to schedule the clearance to improve the performance of an operating aero-engine. The actuators based on ACC management do not execute any action spontaneously when a rub-impact occurs between the rotor and casing, though such events frequently occur [22]. 

Recently, researchers have explored ways of limiting the rub-impact in an aero engine. They have proposed adding damping in the rotating machinery to control the abnormal vibration and reduce the rubbing-induced stress. Ma et al. [23] proposed a multi-objective optimization method to analyze the vibration attenuation effects of the squeeze film damper parameters on the dynamic response of the system. It was found that in the misalignment-rubbing coupling fault, the amplitude of the fundamental frequency reduced by 7.4%, the amplitude of 2× the fundamental frequency dropped by 51.5%, and the amplitude of 3× the fundamental frequency reduced by 16.8%. Their study provided a theoretical reference for vibration control and the optimal design of rotating machinery. Xu et al. [24] suggested an impulsive control method to eliminate the rotor-stator rubbing, based on the phase characteristic. It utilized the vibration energy and the phase difference to trigger the control of the rotor-stator rubbing by impulse. The impulse was applied in directions *x* and *y*, several times, to avoid the rotor-stator rubbing. However, it is a theoretical study, applicable to simple rotors only, and it did not account for the implementation of impulse in practical rotating machinery. Shang et al. [25] investigated the influence of cross-coupling effects on the rubbing-related dynamics of rotor/stator systems. They proposed a control method by generating cross-coupling damping on the stator through the active auxiliary bearing, thereby suppressing the contact severity to avoid rubbing instability. This method was validated by numerical analysis on the Jeffcott rotor model, yet it lacks theoretical analysis and experimental data for complicated rotating systems. However, adding damping to the vibration system is beneficial with minor faults but it is not a valid approach once the rub impact is serious. The research into adding damping in the rotating machinery is not yet well developed.

**Figure 1:**
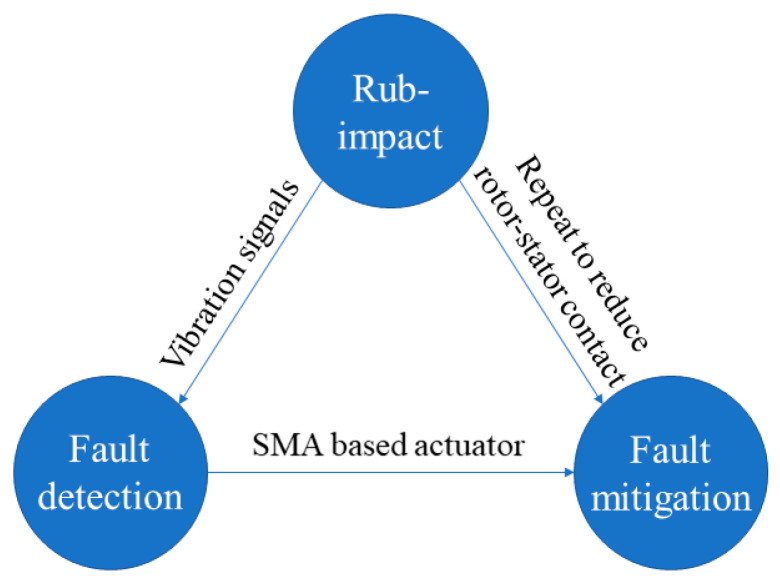
The basic idea of the proposed rub-impact fault mitigation method.

It has been recognized that rub-impact originates from a very small tip clearance between the stationary and rotating parts in an operating aero-engine [26]. If the clearance can be systematically controlled, the rub-impact might be effectively mitigated or eliminated. Garg [27] suggested shape memory alloy (SMA) as an attractive actuation material in an aero-engine due to its high order-of-magnitude energy density and low energy consumption. DeCastro et al. [28] advanced the concept of a prototype actuator consisting of high-temperature shape memory alloys for ACC actuation in the high-pressure turbine section of a modern turbofan engine. Based on the intrinsic properties of SMA and on the development of tip clearance control technology management, this paper presents a method to monitor rub-impact faults and mitigate them via SMA. The proposed method is schematically shown in Figure 1.

This paper presents a promising solution for rub-impact fault detection and mitigation for an aero-engine. An active control scheme, based on two-way SMA wires actuation is proposed. The rub-impact fault is monitored by an acoustic emission (AE) sensor. An SMA wire-based actuator prototype operated by an Arduino control board is established. A series of tests to establish the material properties of NiTi wires, including heating response rate, ultimate strain, free recovery rate, and restoring force, were carried out. The mechanism and design of the actuator are described in detail in this paper. The feasibility of the proposed model for rub-impact fault is verified by our experimental research and our results show that the proposed active control actuator can effectively mitigate rub-impact fault when it occurs. Therefore, the main contribution of this paper is that it provides a potential way to mitigate or even eliminate the accidental rub-impact fault, without a significant reduction in the engine’s efficiency.

## 2. Mechanical Behaviors of SMA Wires

Typical SMA, such as NiTi, is capable of recovering its original shape after plastic deformation by heating above its characteristic transition temperature via the shape memory effect (SME) [29]. This unique mechanical behavior results from a phase transformation between high-temperature austenite and low-temperature martensite phases [30] and has led to many actuation applications [31,32]. In this study, NiTi wires with a two-way shape memory effect [30] were used in our design to drive the actuator through the application of an electric current. As the primary functional component of the actuator, the SME mechanical behaviors of NiTi wires were analyzed in a series of tests prior to design assembly. The material properties of the NiTi wires used in this study and their transition temperatures are listed in Table 1 and Table 2, respectively.

### 2.1. Heating Response Rate of NiTi Wires

**Figure 2:**
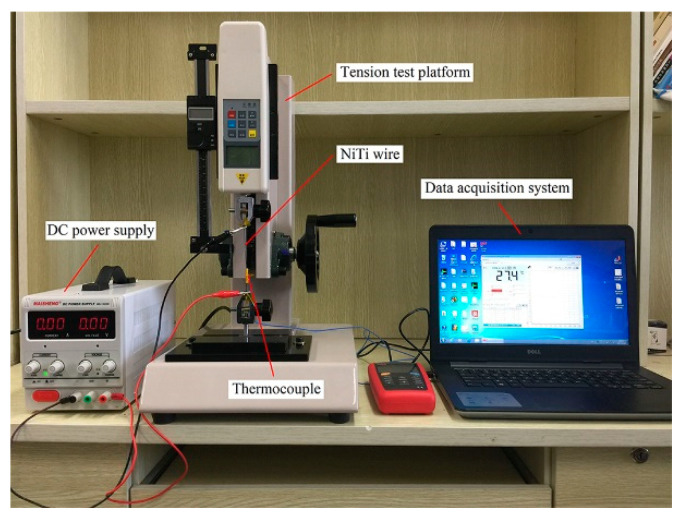
Experimental setup of the heating response rate tests.

The heating response rate of the NiTi wire is used as a measure of how fast the wire’s SME function works. This function is represented by the phase transformation spending time, which is the time it takes the wire to reach its austenite finish temperature A_f_ from its starting state. The shorter the phase transformation spending time, the higher the heating response rate. In order to investigate the heating response rate of NiTi wires, a series of tests under various temperatures were conducted. The experimental setup is schematically shown in Figure 2. The test rig consisted of a DC power supply, a tension test platform, NiTi wire, a thermocouple, and a data acquisition system.

**Figure 3:**
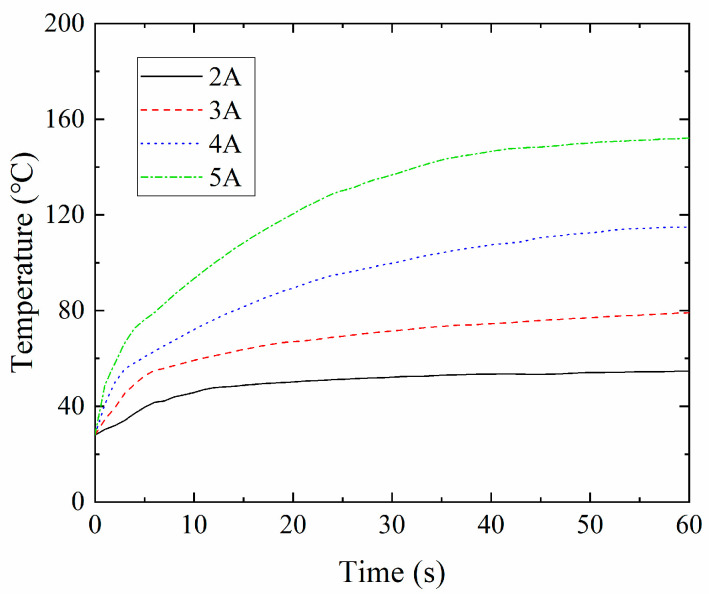
Temperature variation of NiTi SMA wire subjected to different currents.

The length and diameter of the NiTi wire were 100 mm and 0.8 mm, respectively. During the tests, the wire was clamped on a tension test platform and one thermocouple was attached to the wire to measure its surface temperature. At room temperature T_0_ = 21 °C, the wires were heated by 2, 3, 4, and 5 A electric currents provided by the DC power supply. The temperature variations of the wires are shown in Figure 3. The temperature of the NiTi wire rose slowly initially, becoming almost invariable, with an increased electric current. This means that the rising temperature rate of the NiTi wire gradually decreases, and tends to become stable when subjected to a continuous electric current. Figure 3 also indicates that the rising temperature rate of the wire is dependent on the amplitude of the electric current. A large current amplitude is associated with a high rising temperature rate. In order to evaluate the response of the NiTi wires to different electric currents, the phase transformation spending time, which is the time that it takes for the wire to reach A_f_ from its starting state, was measured, using various currents. The comparison results are shown in Table 3. The heating response rate rose with an increased electric current, but an increase in the response rate was not obvious when the electric current increased beyond 6 A.

### 2.2. Ultimate Strain of NiTi Wires

**Figure 4:**
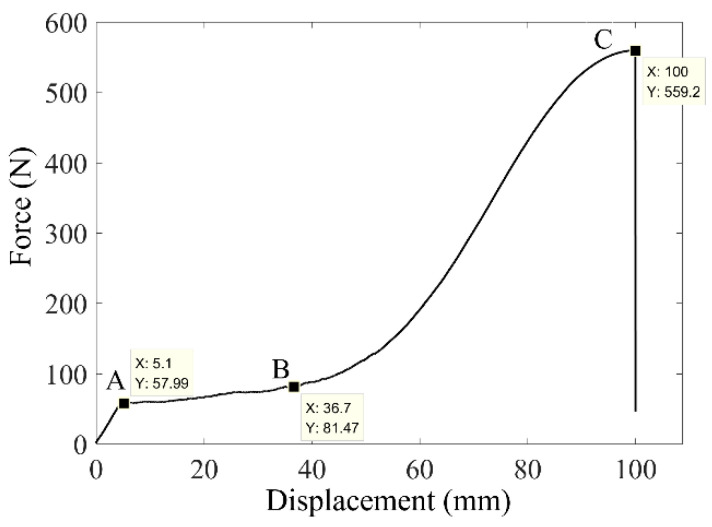
A load-displacement curve under tension test.

A tension test was conducted using an Instron 5565 Universal Testing Machine. The length of the wire specimen used was 750 mm. Because the wire is very fine, it is difficult to accurately measure the ultimate strain directly during its deformation. As such, a load-displacement curve was plotted from the tension test results, as shown in Figure 4. The wire underwent elastic deformation from 0 to point A, plastic deformation from point A to B, a hardening stage from point B to C, and it fractures at point C. The elongations at points A, B, and C are 0.68%, 4.89%, and 13.33% of the original length of the wire, respectively. We found that the NiTi wire behaved well in terms of plasticity and its pre-strain was not allowed to exceed 13.33% due to its ultimate strain.

### 2.3. Free Recovery Rate of NiTi Wires

**Figure 5:**
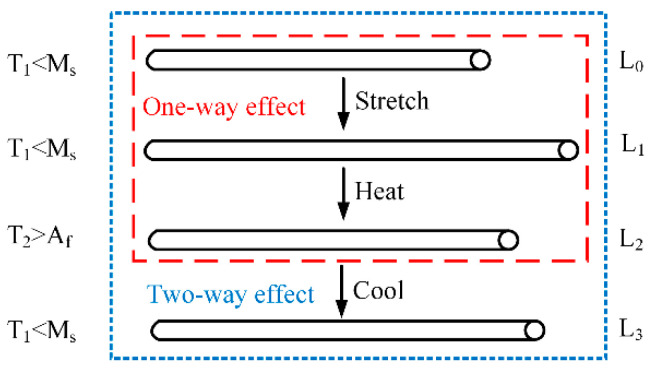
Two-way shape memory effects.

The free recovery rate of NiTi wires under different pre-strain was investigated using the same experimental setup as was used in the heating response rate tests. The initial length of the wire specimens was 100 mm and they were trained to have the characteristic of two-way SME. The wires were stretched with pre-strains of 2%, 3%, 4%, 5%, 6%, and 7%, and were heated to recover their deformations, then cooled to room temperature. As shown in Figure 5, *L*_0_, *L*_1_, *L*_2_, and *L*_3_ represent the original length of the wire, the length of the wire after being stretched and unloaded, the length of the wire after being heated, and the length of the wire after being cooled to room temperature, respectively. The elongations of the wires were measured to calculate the two-way free recovery rate *η*, which is defined by:

(1) $$\eta =\frac{L_{3}-L_{2}}{L_{1}-L_{0}}$$

**Figure 6:**
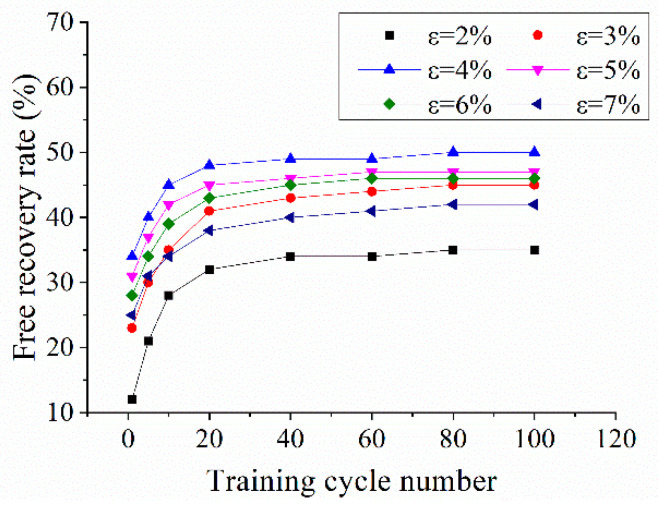
Free recovery rate curve of NiTi wires.

Based on Equation (1), the variation in the free recovery rate with respect to the cycle number at different pre-strain levels is plotted in Figure 6. The two-way free recovery rate initially increased with increased training cycles and then tended to stabilize after 60 cycles. Additionally, the large pre-tension strain resulted in a low free recovery rate. However, the largest free recovery rate appeared when the pre-strain was 4%. Therefore, a median deformation of 4% is preferred in order to obtain the maximum recovery rate of the NiTi wire used in a two-way SME.

### 2.4. Restoring Force of NiTi Wires

**Figure 7:**
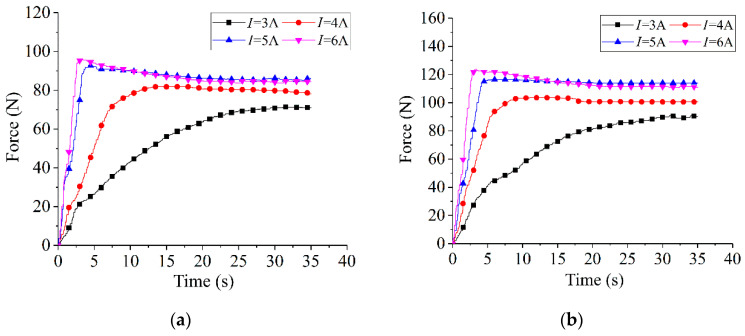

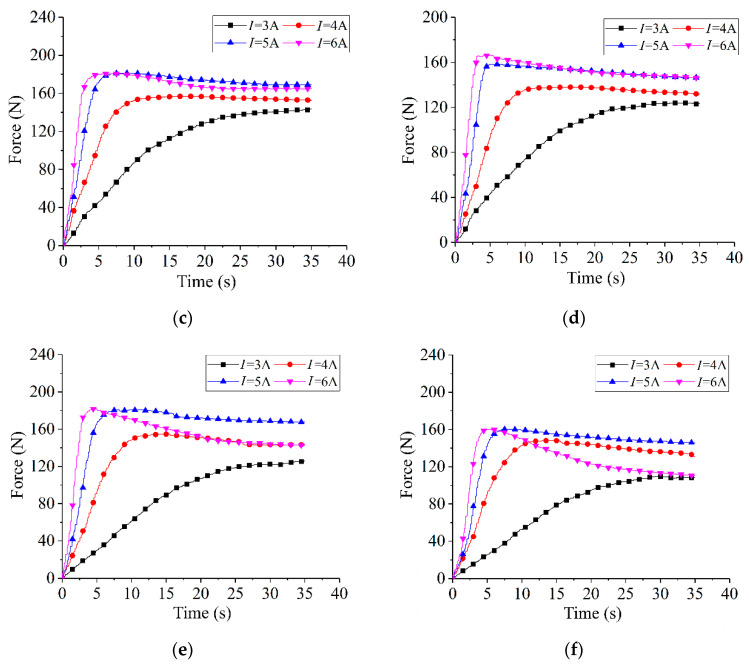
The restoring force of NiTi wires subjected to different electric currents: (**a**) ε = 2%; (**b**) ε = 3%; (**c**) ε = 4%; (**d**) ε = 5%; (**e**) ε = 6%; and (**f**) ε = 7%.

The restoring forces of the NiTi wires, with various pre-strains and which were subjected to 3, 4, 5, and 6 A of electric current, were tested and measured. Figure 7 shows that the restoring force of each wire, at a given electric current, begins to rise sharply and then tends to stabilize. In addition, the maximum restoring force of the NiTi wire with a given pre-strain is dependent on the amplitude of the electric current applied. The restoring force rose with increased current. To compare the heating efficiency of the wires subjected to different electric currents, the response time required to reach the maximum restoring force of each wire was measured and is listed in Table 4. The results indicate that the heating response time to reach the maximum force under a large current does not change significantly. Moreover, the wire with a 4% pre-strain presents the largest restoring force at 6 A. Thus, the designed actuator, installed with a NiTi wire with a 4% pre-strain, may obtain the largest restoring force in an acceptable response time under such conditions.

## 3. Design of the Active Clearance Control Actuator

### 3.1. Prototype of the Actuator

**Figure 8:**
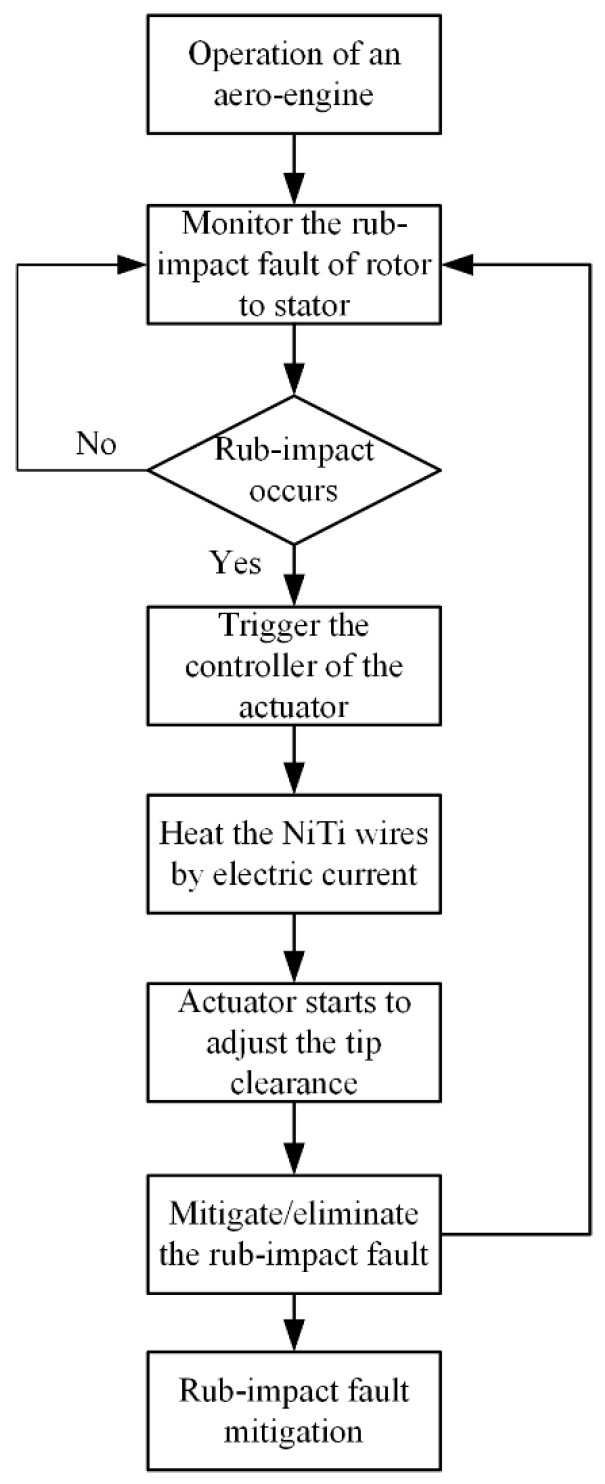
Flowchart of the proposed fault mitigation scheme.

**Figure 9:**
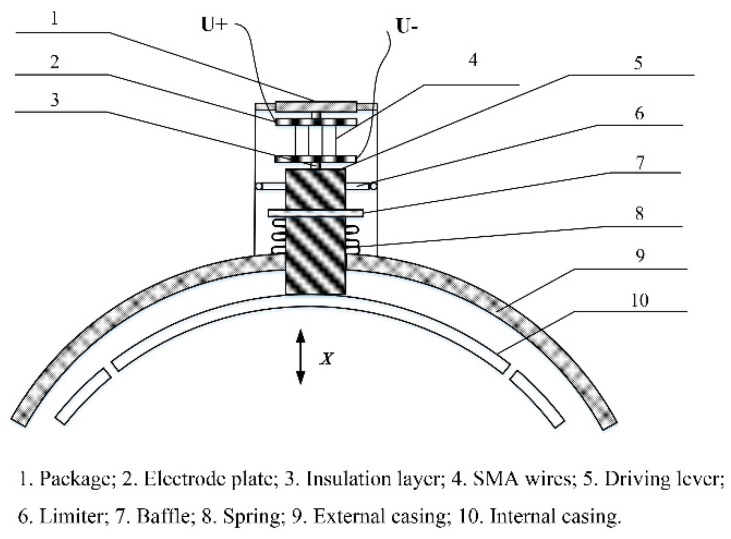
Design of the proposed actuator.

The proposed actuator was designed with the aim of mitigating or eliminating the rub-impact fault when it occurs between the rotor and casing during the operation of an aero-engine. A flowchart of the proposed fault mitigation scheme is shown in Figure 8. The design of the proposed actuator consists of a package, an electrode plate, an insulation layer, pre-stretched NiTi wires, a driving lever, a limit roller, a baffle, a spring, and external and internal casings, as is shown schematically in Figure 9.

**Figure 10:**
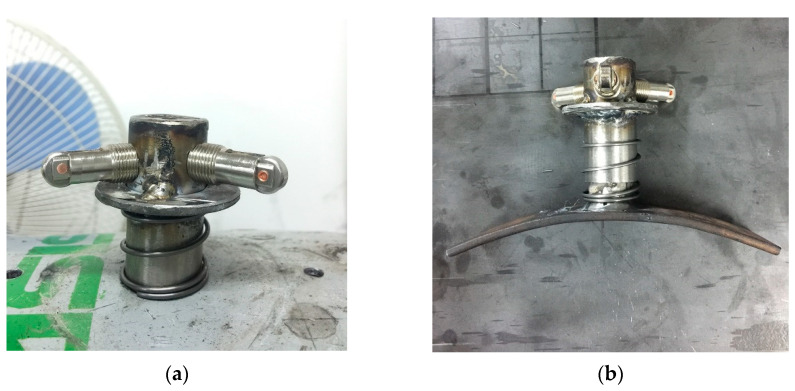

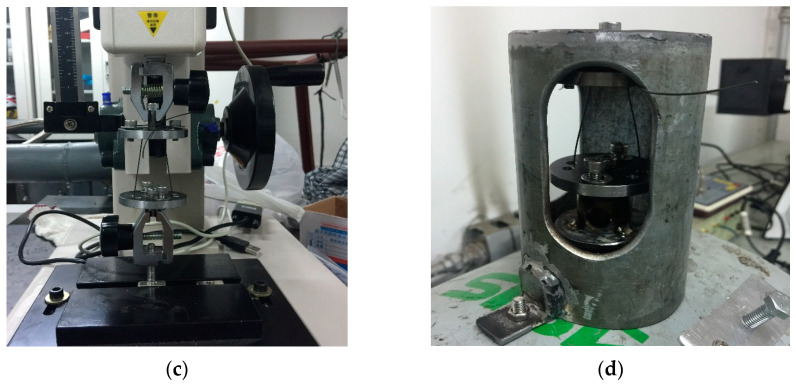
Prototype of the SMA-based actuator: (**a**) Limiter; (**b**) driving lever and internal casing; (**c**) electrode plates; and (**d**) package.

The actuator works by using a driving lever that passes through the external casing. The two ends of the lever are fixed on the internal casing and the bottom electrode plate, respectively, which forces the internal casing to deform when the lever moves. Two insulation layers are placed between the electrode plates and the other parts of the actuator for the insulation and protection of the entire structure. The two ends of NiTi wires are bolted at each end to two electrode plates and are heated by an electric current during the operation of the actuator. A baffle is welded onto the driving lever and a spring is installed between the baffle and the external casing to generate a restoring force. In addition, one limiter with three rollers, circumferentially threaded and installed on the driving lever, act to improve the accuracy of the motion. The prototype of the actuator is shown in Figure 10. To allow us to observe the behavior of the actuator, the package was not completely sealed, as shown in Figure 10d.

Once a rub-impact fault is detected, the external direct current (DC) power supply system begins to heat the SMA wires. When the temperature is beyond the austenite start temperature of the wires A_s_, the wire undergoes a martensite phase transformation and is compressed due to the unique property of SME. This compression forces the internal casing to move upwards via the driving lever. Due to the motion of the internal casing, the tip clearance between the rotor and the casing is enlarged and, as a result, the rub-impact phenomenon is mitigated or possibly eliminated. The aim of using two-way NiTi wires is to mitigate the rubbing fault, whilst guaranteeing engine efficiency, which is achieved when the clearance is reduced once the NiTi wires cool to the martensite phase. To verify the feasibility of the proposed model, a prototype of an SMA-based actuator was designed and its geometric parameters are shown in Table 5.

### 3.2. Control Scheme of the Actuator

**Figure 11:**
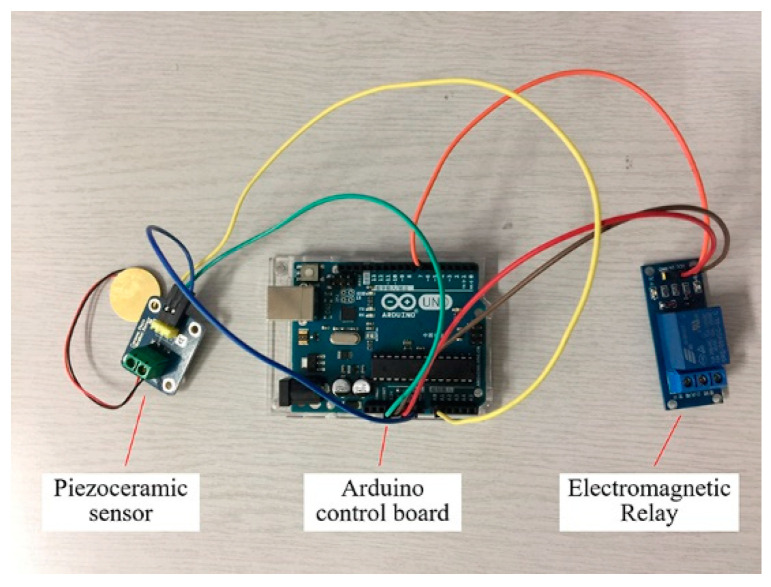
Configuration of the control system.

**Figure 12:**
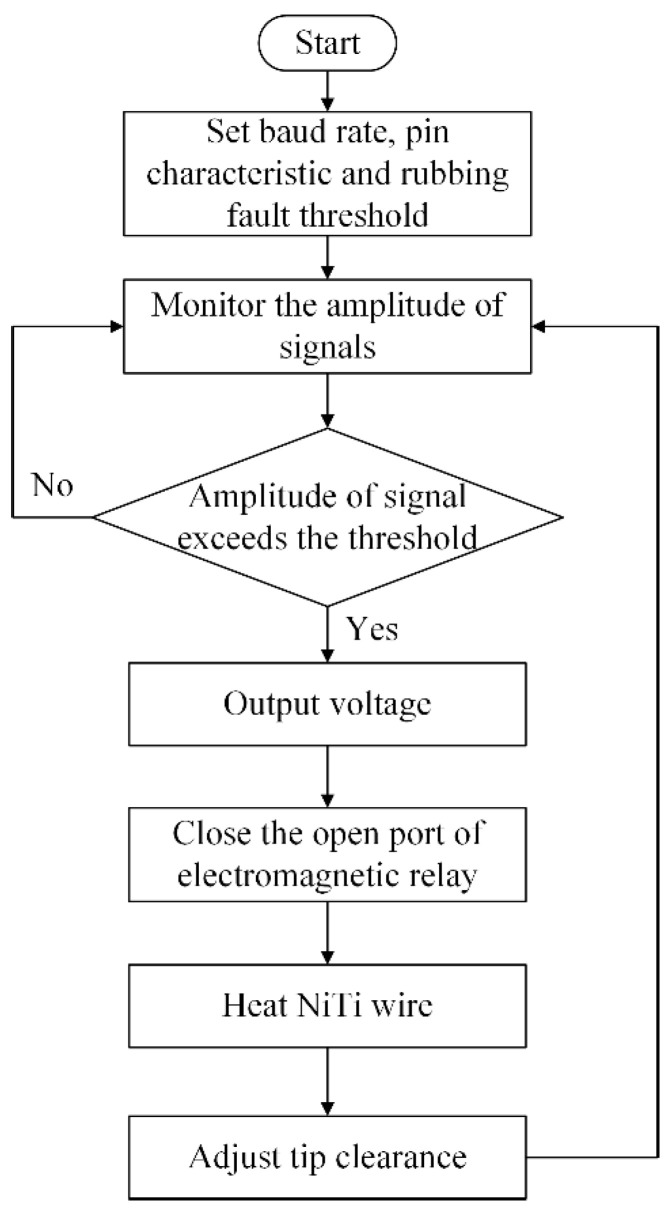
Flowchart of the control scheme.

In order to realize the self-healing of rub-impact faults, a control system based on an Arduino control board was investigated for the proposed actuator. This system consists of an Arduino Uno R3 control board, an RB-02S082A piezoelectric sensor, and an SRD-05VDC-SL-C 5V electromagnetic relay, as shown in Figure 11. When the system is running, the AE signals are acquired by the piezoelectric sensor and are then transferred to the Arduino Uno R3 control board, which is capable of identifying whether the acquired signals are fault signals. The electric circuit switching function is implemented by compiled coding. In normal conditions, the current circuit is shut down and the actuator does not work. Once a rub-impact fault is identified by the board, an order is sent to the electromagnetic relay to switch on the current circuit to heat the NiTi wires. A flowchart depiction of the algorithm of the control scheme is shown in Figure 12. 

## 4. Experimental Verification of the Clearance Control Mechanism

**Figure 13:**
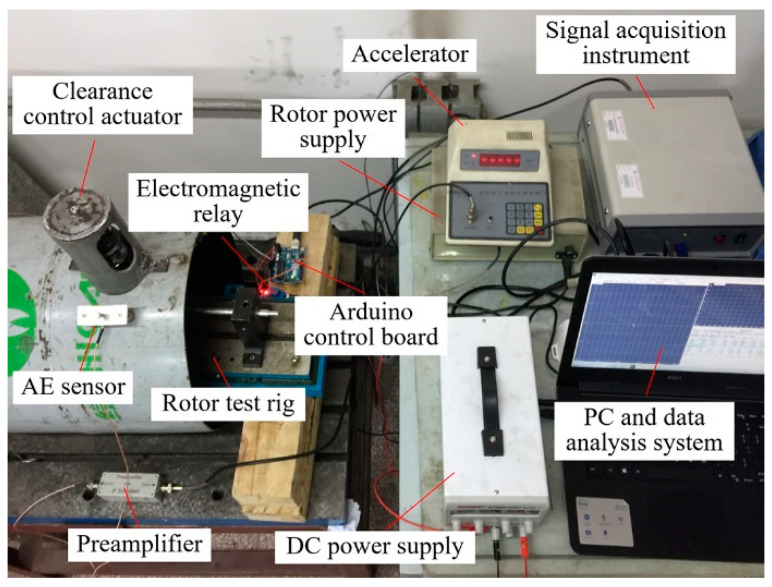
Equipment of the clearance control mechanism.

In order to evaluate the effectiveness of the clearance control actuator, the mechanism was verified by an experimental study. Figure 13 shows the experimental setup, which consisted of a rotor test rig, a rotor power supply, an accelerator, an electromagnetic relay, an Arduino control board, a piezoelectric sensor, an AE sensor, a preamplifier, a signal acquisition instrument, a DC power supply, and a PC and data analysis system. 

The NiTi wires with 4% pre-strain were installed on the clearance control actuator, and the actuator was fixed on the external casing of the aero-engine. The power of the rotor test rig was provided by a rotor power supply and the rotational speed of the rotor was controlled by an accelerator. The piezoelectric sensor was attached to the upper surface of the internal casing and was connected to the Arduino control board to identify the rubbing and control the electromagnetic relay operation. An AE sensor was attached to the surface of the external casing to acquire the AE signals. These AE signals were processed by a signal preamplifier and then transmitted to the PC.

### 4.1. Setting of the Threshold and Sampling Frequency

**Figure 14:**
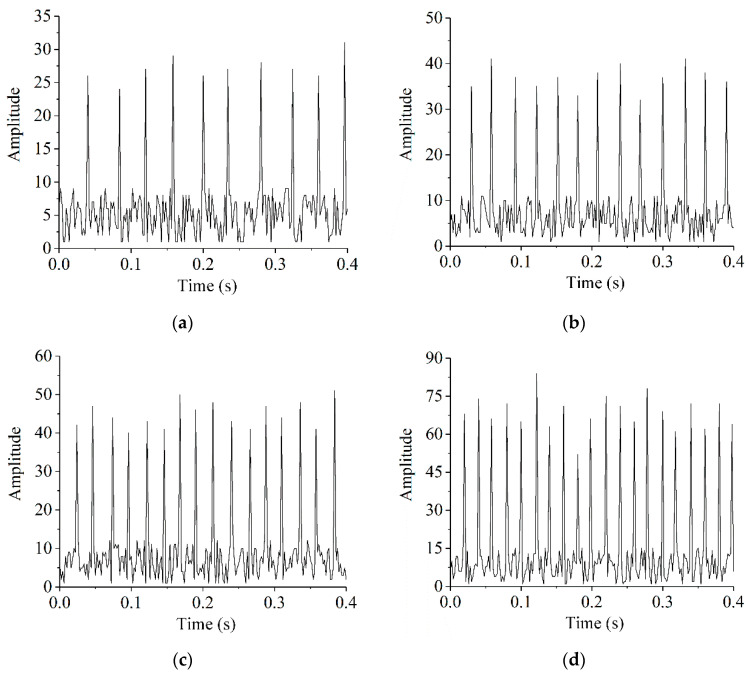
Vibration signal at various rotational speeds: (**a**) *n* = 1500 rpm; (**b**) *n* = 2000 rpm; (**c**) *n* = 2500 rpm; and (**d**) *n* = 3000 rpm.

The accuracy of the proposed control scheme is dependent on the selection of the signaling threshold and sampling frequency. The operation of an aero-engine is always accompanied by vibrations, whether or not rub-impact occurs. Therefore, the primary goal is to ascertain the appropriate threshold at which rub-impact faults occur, by reference to the amplitude of the vibration signal. Figure 14 shows the signals acquired by the piezoelectric sensor at rotational speeds of 1500, 2000, 2500, and 3000 rpm. The amplitudes of all vibration signals without the rub-impact fault are lower than 20. Therefore, the threshold may be set to 20 as the threshold for the occurrence of the rub-impact fault. Regarding the sampling, in our experience, it is generally better for the set to be greater than 5 times the highest frequency of the vibration signals, to ensure the reliability of the acquired data. Because the rotational frequency of the rotor at 3000 rpm was measured at 50 Hz in our tests, the sampling frequency in this study was set to 500 Hz, which was 10 times the highest frequency.

### 4.2. Verification of the Clearance Control Actuator

(1) Effect of rotational speed

The effectiveness of the proposed clearance control actuator was verified at rotational speeds of 1500, 2000, 2500, and 3000 rpm, with a 6 A electric current, in our tests. Assuming that *t*_0_, *t*_1_, *t*_2_, *t*_3_, *t*_4_, *t*_5_ represent the points at which a rub-impact fault occurs, the amplitude of the AE signal reaches its maximum, the fault is eliminated, the second rub-impact fault begins, the amplitude of the AE signal reaches its maximum again, and the second fault is eliminated, consecutively. The specific durations are defined by:

(2) $$T_{1}=t_{1}-t_{0}$$

(3) $$T_{2}=t_{2}-t_{1}$$

(4) $$T_{3}=t_{3}-t_{2}$$

(5) $$T_{4}=t_{4}-t_{3}$$

(6) $$T_{5}=t_{5}-t_{4}$$

where *T*_1_ is the duration from the start of rub-impact to its most serious state, *T*_2_ denotes the rub-impact fault elimination time spent by the proposed actuator, *T*_3_ represents the time interval between the rub-impact fault and the start of the following rub-impact fault event, *T*_4_ is the duration from the start of the next fault to its most serious state, and *T*_5_ denotes the time spent to eliminate the fault. 

Table 6 gives the spending times measured at various rotational speeds in the rotation test, at a 6 A electric current. The results show that the control times *T*_1_, *T*_2_, *T*_3_, *T*_4_, and *T*_5_ are almost the same at different rotational speeds. This means the control time of the clearance control mechanism based on two-way NiTi wires is independent of the rotational speed of the rotor.

**Figure 15:**
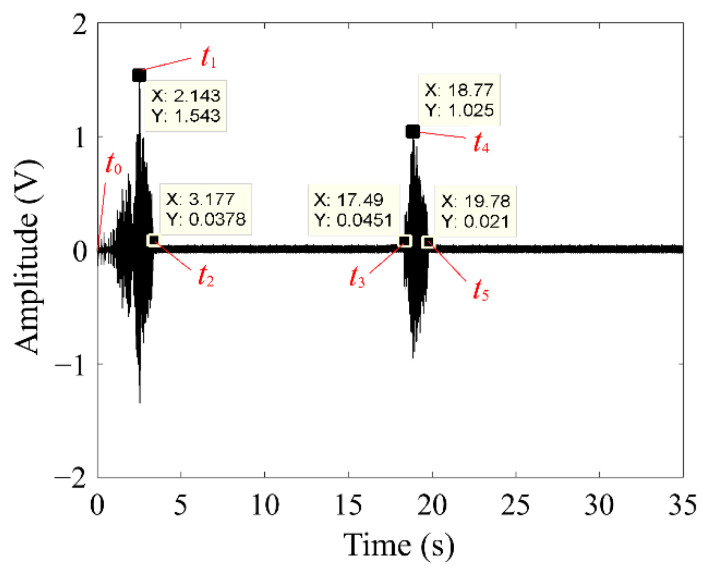
AE signals acquired during the clearance control process based on two-way NiTi SMA wires.

A series of rotational rubbing tests were conducted under the condition of a 6 A current and a 1500 rpm rotational speed, and the AE signal during the operational process was measured. As shown in Figure 15, the amplitude of the AE signal continuously rises with increased rotor speed during the start-up process until it reaches the maximum at *t*_1_ = 2.143 s, i.e., the rubbing becomes serious and a fault at this moment. At *t*_2_ = 3.177 s, this rubbing fault is eliminated. The next rubbing fault occurs at *t*_3_ = 17.49 s, becoming severe at *t*_4_ = 18.77 s. At *t*_5_ = 19.78 s, the fault is eliminated.

(2) Effect of the electric current

A series of rotational rubbing tests at 4, 5, and 6 A electric currents were conducted at rotational speeds of 1500 rpm. The rubbing fault eliminating time spent during the control process is recorded in Table 7. It was found that *T*_2_, *T*_3,_ and *T*_5_ changed significantly with different electric currents. The durations of *T*_2_ and *T*_5_ gradually descended with increased current. Therefore, a large electric current contributes to a rapid control response time. In addition, the interval between one rub-impact fault and the next fault, *T*_3_, becomes larger at a higher current compared to lower currents, resulting from the fact that the NiTi wires need more time to cool. The proposed actuator is affected significantly by the electric current. 

## 5. Conclusions

A fault mitigation scheme based on a two-way SMA wire system to mitigate and possibly eliminate the rotor-stator rub-impact of an aero-engine, by controlling the tip clearance between the rotor and stator, was proposed in this study. Based on the inherent characteristics of SMA, a prototype of an SMA-based actuator was designed, manufactured, and tested. Through our experimental study, the proposed scheme was verified and the following conclusions were drawn:

(1) The mechanical properties of NiTi wire are highly dependent upon the heating electric current and on its pre-strain level. It is recommended to apply a 6 A electric current to heat the wires and to use wires with a 4% pre-strain in the design of an SMA wire-based actuator.
(2) The time spent in the fault mitigation/elimination process is not significantly dependent on the rotational speed of the rotor.
(3) The proposed SMA-based actuator is a promising model that mitigates the rub-impact fault that occurs between the rotor and stator in aero-engines, whilst guaranteeing engine efficiency.

## Figures and Tables

**Table 1:** Material properties of NiTi wires.

Name	Composition	Density (Kg·m^−3^)	Melting Point (°C)
NiTi	50.3 at%Ni	6500	1295

**Table 2:** Phase transition temperatures of NiTi wires.

M_f_ (°C)	M_s_ (°C)	A_s_ (°C)	A_f_ (°C)
19.8	36.3	39.7	50.1

**Table 3:** The phase transformation spending time to reach A_f_ using different currents.

I (A)	2	2.5	3	3.5	4	5	6	7	8
**t (s)**	14.9	6.3	3.2	1.9	1.5	1.1	0.7	0.4	0.3

**Table 4:** Maximum restoring force and required time at different electric currents.

Strain (%)	Electric Current (A)	Maximum Restoring Force (N)	Time to Reach the Maximum Restoring Force (s)
2	3	71.3	31.2
	4	81.8	16.3
	5	92.7	4.2
	6	95.8	3.1
3	3	90.6	31.0
	4	103.5	11.1
	5	116.4	5.6
	6	123.3	3.1
4	3	142.4	32.8
	4	156.7	16.5
	5	181.0	7.1
	6	180.1	5.0
5	3	123.7	30.7
	4	138.0	15.9
	5	158.4	4.9
	6	165.9	3.3
6	3	124.6	33.2
	4	154.7	13.7
	5	181.0	7.6
	6	181.8	4.4
7	3	109.6	29.8
	4	148.0	13.6
	5	159.5	6.9
	6	159.3	4.7

**Table 5:** Design of the proposed actuator prototype.

Parts	Shape	Dimensions
Package	Hollow cylinder	Internal diameter: 70 mm External diameter: 76 mm Height: 150 mm
Electrode plate	Disc	Diameter: 40 mm Thickness: 3 mm
NiTi wire	Wire	Length: 50 mm Diameter: 0.8 mm Pre-strain: 4%
Driving lever	Rod	Diameter: 26 mm Height: 70 mm
Spring	Compression spring	Internal diameter: 28 mm Free height: 25 mm Wire diameter: 1.5 mm
Limiter	Hollow cylinder with three rollers on the side of the cylinder	Internal diameter of cylinder: 70 mm External diameter of cylinder: 76 mm Height of cylinder: 150 mm External diameter of roller: 6 mm Thickness of roller: 2 mm

**Table 6:** Control time of the clearance control actuator at different rotational speeds.

*I* (A)	*n* (rpm)	*T*_1_ (s)	*T*_2_ (s)	*T*_3_ (s)	*T*_4_ (s)	*T*_5_ (s)
6	1500	2.1	1.0	14.3	1.3	1.0
6	2000	1.8	0.9	13.5	1.2	0.8
6	2500	1.8	1.1	14.0	1.4	0.7
6	3000	2.1	1.0	13.7	1.3	1.1

**Table 7:** Control time of the NiTi wire based actuator at various electric currents.

*I* (A)	*n* (rpm)	*T*_1_ (s)	*T*_2_ (s)	*T*_3_ (s)	*T*_4_ (s)	*T*_5_ (s)
4	1500	2.3	13.6	7.2	1.1	13.1
5	1500	2.0	4.7	10.4	1.1	4.4
6	1500	2.1	1.0	14.3	1.3	1.0

## Data Availability

Not applicable.

## Abbreviations

The following abbreviations are used in this manuscript:

SMA	Shape memory alloy
AE	Acoustic emission
ACC	Active clearance control
SME	Shape memory effect
DC	Direct current
